# *p*-Xylene Oxidation to Terephthalic Acid: New Trends

**DOI:** 10.3390/molecules28041922

**Published:** 2023-02-17

**Authors:** Hugo M. Lapa, Luísa M. D. R. S. Martins

**Affiliations:** 1 Centro de Química Estrutural, Institute of Molecular Sciences, Departamento de Engenharia Química, Instituto Superior Técnico, Universidade de Lisboa, Av. Rovisco Pais 1, 1049-001 Lisboa, Portugal; 2 Departamento de Engenharia Química, Instituto Superior de Engenharia de Lisboa, Instituto Politécnico de Lisboa, R. Conselheiro Emídio Navarro 1, 1959-007 Lisboa, Portugal

**Keywords:** *p-*xylene, terephthalic acid, oxidation, polyethylene terephthalate

## Abstract

Large-scale terephthalic acid production from the oxidation of *p*-xylene is an especially important process in the polyester industry, as it is mainly used in polyethylene terephthalate (PET) manufacturing, a polymer that is widely used in fibers, films, and plastic products. This review presents and discusses catalytic advances and new trends in terephthalic acid production (since 2014), innovations in terephthalic acid purification processes, and simulations of reactors and reaction mechanisms.

## 1. Introduction

**Figure 1:**
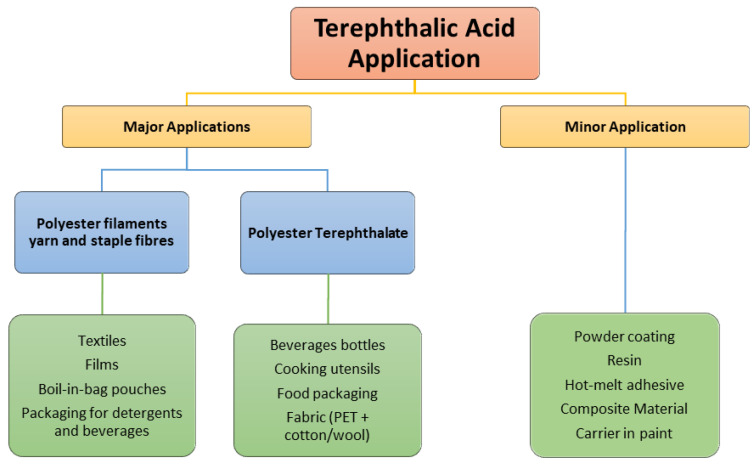
Applications of terephthalic acid (1,4-benzenedicarboxylic acid, TPA). Adapted from [1].

The main and most important product of *p-*xylene oxidation is terephthalic acid (1,4-benzenedicarboxylic acid, TPA), the main component in the polyester industry for the production of polyester terephthalate, commonly known as PET. Although the production of PET uses a great proportion of the terephthalic acid produced worldwide, terephthalic acid has other applications such as in textiles, in polyester staple fibers and filament yarns, as a carrier in paints, a coating resin, and as a raw material in the pharmaceutical industry (Figure 1).

The current worldwide purified terephthalic acid market was evaluated to be between USD 51.5 and 54.8 billion in 2021, and is estimated to reach USD 70 or 78 billion at the end of the decade [2,3,4]. This would represent a compound annual growth rate of between 4.6 and 5.6%. Globally, there is an installed capacity of ca. 81.6 Mmt per year, and this is estimated to increase to 105.6 Mmt per year by the end of 2029 [5].

### 1.1. The Beginning of Terephthalic Acid Production

The story of and need for terephthalic acid are directly linked to the production of polyester and have been the driving force for changes and intensive research in this field.

**Scheme 1:**
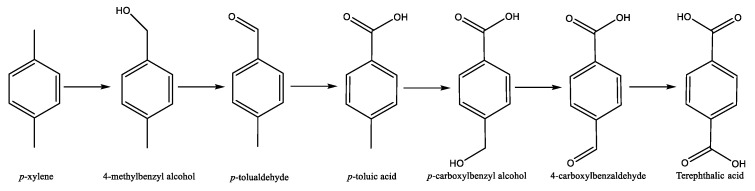
Reaction pathway and products obtained for the oxidation of *p-*xylene.

Petroleum-based terephthalic acid uses *p*-xylene (produced via the fractional distillation of naphtha) as a raw material (Scheme 1).

The first report on the oxidation of *p-*xylene was produced in 1912 by Ciamician and Silber [6]. This study evaluated the effect of sunlight and the presence of molecular oxygen, used as an oxidant, for a year. In the end, the result showed the presence of terephthalic acid among the products, in addition to *p-*toluic acid. Later, in 1926, Stephens [7] contributed to the understanding of the oxidation mechanism by disclosing the pathway that the reactants (hydrocarbon and alkane) undergo in a stepwise manner, and showed that aldehydes were intermediates for this reaction. Although both studies were starting to produce terephthalic acid, this would not have a practical industrial application due to the long reaction time required. Therefore, the use of homogeneous or heterogeneous catalysts was mandatory.

One of the first industrial and commercially viable routes to producing terephthalic acid was the liquid phase oxidation of *p-*xylene [8] using diluted nitric acid (30–40%) as an oxidant, applying temperatures between 160 and 200 °C and pressures from 8.5 to 13.5 bar. The setup presented an explosive, hazardous environment and the terephthalic acid produced was contaminated with colored impurities. Thus, the method was replaced by the Dynamit Nobel process [9], which exchanged the hazardous nitric oxidation route for the solvent-free air oxidation of *p-*xylene (with temperatures between 140 and 180 °C, and pressure between 5 and 8 bar) using cobalt as a catalyst. In this process, *p-*xylene was first oxidized to *p-*toluic acid, which was then esterified by methanol. In the end, *p-*toluate was oxidized by air to monomethyl terephthalate, which was esterified with methanol, leading to dimethyl terephthalate (DMT). It is worth highlighting that, in PET’s first years, its polymers were all made from DMT. The Dynamit Nobel solution allowed researchers to overcome the resistance of *p-*toluic acid to oxidation but came with an increased operating cost due to the many steps required in esterification by methanol. It ended with commercialization of the AMOCO process [10,11,12] in the late 1970s.

Currently, the most widespread technology for producing terephthalic acid is the Mid-Century process, also known as the AMOCO process, which consists of the direct liquid phase catalytic oxidation of *p-*xylene with air in the presence of a transition-metal catalyst, usually in a homogeneous reaction medium. This process depends on the operating conditions and catalysts used (see below).

### 1.2. The AMACO Process

The resistance of *p-*toluic acid to oxidation was first discovered and overcome in 1955 by the Mid-Century Corp [10,11,12] and ICI. The process was developed by Standard Oil, Indiana, and later, AMOCO, with some input from ICI. In the AMOCO process, the oxidation of *p-*xylene occurs in the liquid phase using acetic acid as solvent, oxygen as an oxidant, and a combination of three ions as homogeneous catalysts: cobalt, bromide, and manganese. The reactor operates at temperatures of ca. 175–225 °C and at pressures of 15–30 bar. The metallic ion source can be obtained from various salts of cobalt and manganese, preferably the acetate metal salts, while hydrobromic acid, sodium bromide, or tetrabromoethane can be used as a bromide ion source. It is preferable to use the first option. During the reaction, the terephthalic acid that is formed ends up in a solid form due to its low solubility in acetic acid. Overall, the reaction achieves conversion of more than 98% of *p-*xylene with ca. 95% selectivity towards terephthalic acid between 8 and 24 h. Although high conversion and selectivity are obtained, the pure terephthalic acid should not have more than 25 ppm of 4-carboxybenzaldehyde (4-CBA); therefore, purification of the crude terephthalic acid must be performed after the reaction, by dissolving the crude terephthalic acid in hot water in the presence of a palladium catalyst, to reduce the 4-CBA to *p-*toluic acid [13].

The last intensive review of this topic was published in 2013, and since then, several important studies and reports have been published throughout the years [14]. Therefore, this review will summarize the new advances that have emerged since 2014.

### 1.3. Production from Biomass and Availability of p-Xylene

The use of biomass as a source for chemical production is a “hot topic” and has been the subject of study and review in recent years: While the reaction conditions are substantially cleaner, avoiding the use of bromides and acetic acid, the pathway is long, including several different reactions. [15,16] Another major feature is the availability of pure *p-*xylene from the other isomers. As demonstrated by Wang et al. [17], the production of *p-*xylene is still a challenge. Most catalytic systems tend to perform poorly when applied to the other isomers, and there is also a need to address the difficulty of separation. [18] A final major positive point is the possibility of bypassing toluic acid during synthesis, one of the hardest compounds to oxidize during the process.

## 2. Catalytic Advances

### 2.1. Homogeneous Catalysts

The liquid phase oxidation of *p-*xylene is very promising. As described above, the AMOCO process is a homogeneous catalytic system that, despite having excellent yields, utilizes acetic acid as a solvent and bromide compounds, such as hydrobromic acid (HBr) or sodium bromide (NaBr); this creates a hazardous reaction environment that is highly corrosive, and bromide compounds are not only non-environmentally friendly, but also harmful and dangerous to handle.

Therefore, research has been conducted to discover new catalytic systems that are less corrosive and more environmentally friendly for the oxidation of *p-*xylene. To better understand the developments that took place before this review, these articles summarizes what has been publish in the field of the homogeneous catalysis of *p-*xylene. [1,14,19].

In 2014, Plekhov et al. [20] studied the possibility of oxidizing *p-*xylene to terephthalic acid using molecular oxygen as an oxidant with acetate salts of cobalt(II) and manganese(II) in the presence of *N-*hydroxyphthalimide (NHPI), and using acetic acid as a solvent. As reported before, the catalytic systems of NHPI-Co(II) have a good synergistic effect; it is therefore interesting to study this reaction. Without Mn(II)’s presence, the reaction performed at 65 °C for 3 h led only to the intermediate products *p-*tolualdehyde and *p-*toluic acid. However, the initial reaction rate and conversion were higher than when the reaction was performed in the presence of a cobalt(II) and manganese(II) bromide salt catalysts. Upon adding manganese acetate as a catalyst, the authors observed a slight increase in conversion (35–40%), selectivity for *p-*toluic acid (85–89%), and oxidation rate (4.1–4.6 × 10^4^ mol L^−1^ s^−1^). Since *p-*toluic acid is known to have an electron withdrawal effect on the methyl group at the *p*-position, Plekhov tested the same system at 90 °C for the oxidation of *p-*toluic acid. Much smaller conversion was obtained when compared to the oxidation of xylene: 12% over 3 h, but with selectivity of terephthalic acid of 93%.

**Figure 2:**
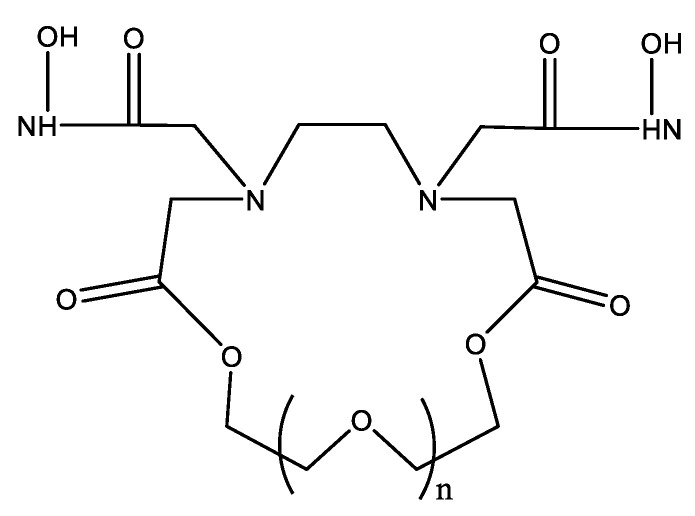
Example of the aza-crowned dihydroxamic acid, *n* = 0–4. Adapted from [21].

Later in the same year, Wei et al. [21] reported the synthesis and application of new cobalt(II) aza-crowned dihydroxamic acid complexes (Figure 2). The oxidation reaction occurred in gas–liquid apparatus, where liquid *p*-xylene was oxidized by air bubbled into the mixture at a flow rate of 2.0 L min^−1^, at 110 °C, for a maximum of 5 h. Precipitates were formed over longer durations. Interesting information about this type of catalyst can be extracted from the results obtained: on one hand, the size of the crown ether ring enhanced the catalytic performance of the complexes, creating a small space favorable for the substrate; on the other hand, the steric hindrance of the crown ring shielded the active metal center, leading to a disadvantageous configuration to the formation of active oxygen species and their interaction with the substrate. Catalytically, the best result was obtained after an induction period of 0.3 h and an oxidation time of 5 h: *p-*xylene conversion achieved 84.8% with a yield of *p-*toluic acid of 80.2%, representing selectivity of 94.6%.

In 2016, Wang and Tong [22] reported the production of *p-*xylene and terephthalic acid through the bio-based conversion of isoprene and acrolein. During the process of the production of *p-*xylene, an oxidized product was obtained: 4-methylbenzaldehdyde. Since this “by-product” is part of the reaction route of producing terephthalic acid, its oxidation was investigated. The reaction could have been performed using KMnO_4_, but due to its toxicity and environmental impact, an alternative path was taken using cobalt(II) acetate and manganese(II) acetate and NHPI in acetic acid, under oxygen, at reflux and atmospheric pressure. After 14 h of refluxing, a final yield of 91% of TPA was obtained, and the overall yield of all processes using biomass achieved 32%.

**Figure 3:**
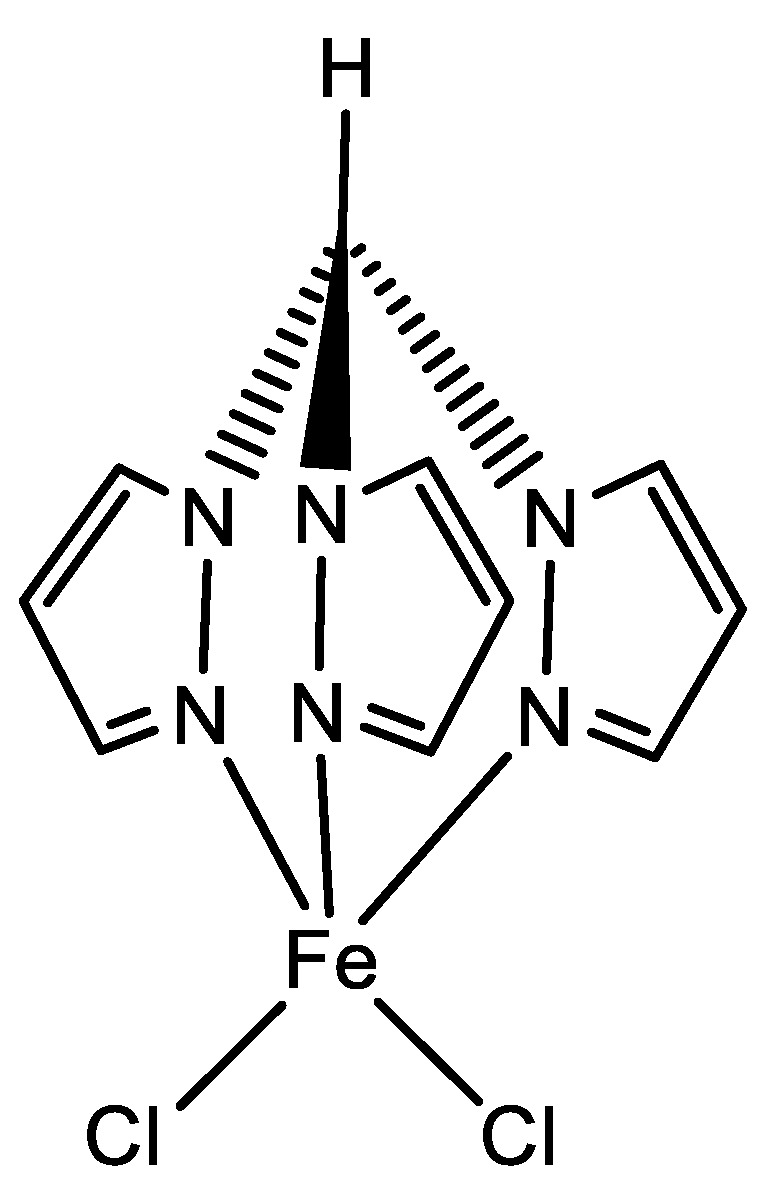
Iron(II) C-scorpionate complex structure. Adapted from [23].

**Scheme 2:**
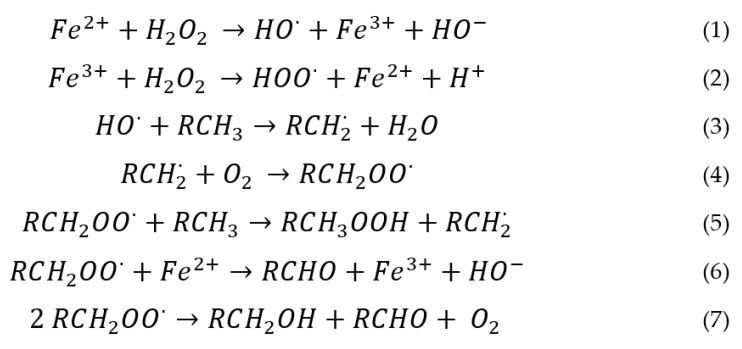
Proposed mechanism for the oxidation of *p-*xylene catalyzed by a C-scorpionate iron(II) complex. Adapted from [23].

C-scorpionate complexes are known for their activity in alkane oxidation. In 2016, Mendes et al. [23] reported the use of an iron(II) C-scorpionate complex (Figure 3) for the oxidation of *p-*xylene at low temperatures and in the presence of 30% H_2_O_2_. The most impressive result occurred with 10 µmol of catalyst in acetonitrile, nitric acid in a ratio [n(acid)/n(catalyst)] of 10, at 35 °C. After only 5 min, a total yield of 21.8% of oxygenated products, representing a TOF of 1.3 × 10^2^ h^−1^, was formed. However, the main product was *p-*tolualdehyde, far from the last oxidation product of TPA (see Scheme 2). *m*- and *o*-xylene were also tested. The presence of nitric acid improved the reaction yield of the aldehyde formation, but the highest value was found for *p*-xylene, probably due to steric limitations that can exist with the *ortho*- and *meta*- positions. The mechanism for the reaction with this type of catalyst is thought to be a free-radical mechanism due to the monofunctional product and lack of ring hydroxylation (Scheme 2). The changes in the oxidation state of the metal center (+2/+3) detected by XPS also support the authors’ hypothesis.

**Figure 4:**
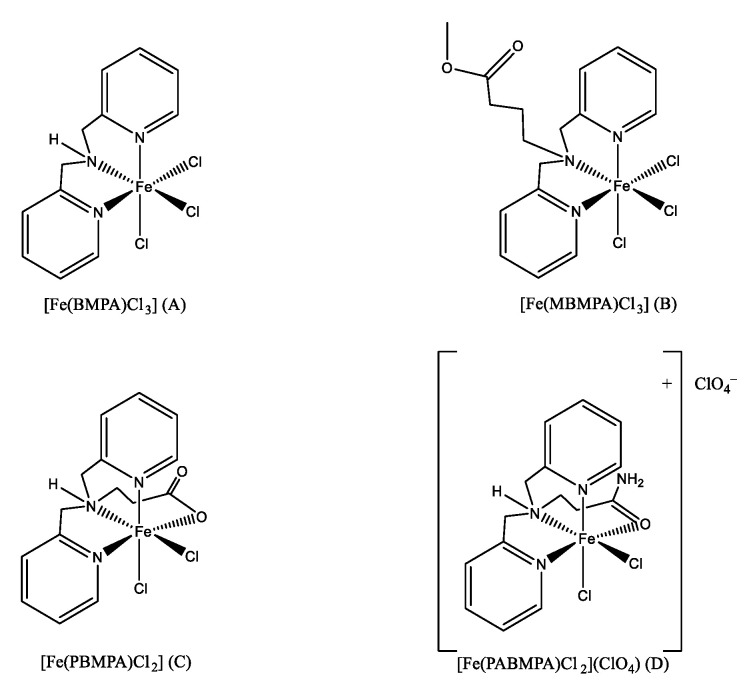
Structures of iron(III) containing bis-(2-pyridymethyl)amine) (BMPA) and derivatives. Adapted from [24].

Inspired by enzymes such as methane monooxygenase and toluene 4-monooxygenase, Antunes and co-workers [24] reported non-heme iron(III) complexes bearing bis-(2-pyridylmethyl)amine (BMPA) and derivatives (Figure 4) as catalysts for the selective oxidation of aromatic compounds by H_2_O_2_. The authors tested the most active catalyst for the oxidation of toluene [Fe(BMPA)Cl_3_], with several other aromatic compounds, including *p-*xylene, with the following conditions: 50 °C, 24 h, 0.77 mol L^−1^ of substrate, 7.7 × 10^−3^ mol L^−1^ of catalyst, and H_2_O_2_ (0.77 mol L^−1^). The result obtained for the xylene oxidation was in line with that obtained with toluene, and the main products for both reactions were, respectively, 2,5-dimethyl-2,5-cyclohexen-1,4-dione and cresol (mainly *o*-cresol). This shows that preferable oxidation occurs on the aromatic ring instead of the methyl groups. The authors explained that such a result can be associated with the presence of hydroxyl radicals, formed via a radical mechanism through the autoxidation process with a highly electrophilic oxo-metal transient species reacting with the arene π-system. Another interesting aspect of this study is the fact that for toluene oxidation, the increase in temperature (25 to 50 °C) increased the selectivity for benzaldehyde, meaning that the selectivity for methyl oxidation increased. Since only a study at 50 °C was performed for *p*-xylene, it would be interesting to compare the result with lower and higher temperatures to see if the same behavior is observed. In the end, *p*-xylene oxidation led to a total yield of 16% with 42% selectivity for the main product, resulting from oxidation of the aromatic ring. The other complexes (Figure 4B–D) were not tested for the oxidation of *p*-xylene and were only tested of the oxidation of toluene, whereby the authors obtained similar or lower yields when compared to the other complex.

**Figure 5:**
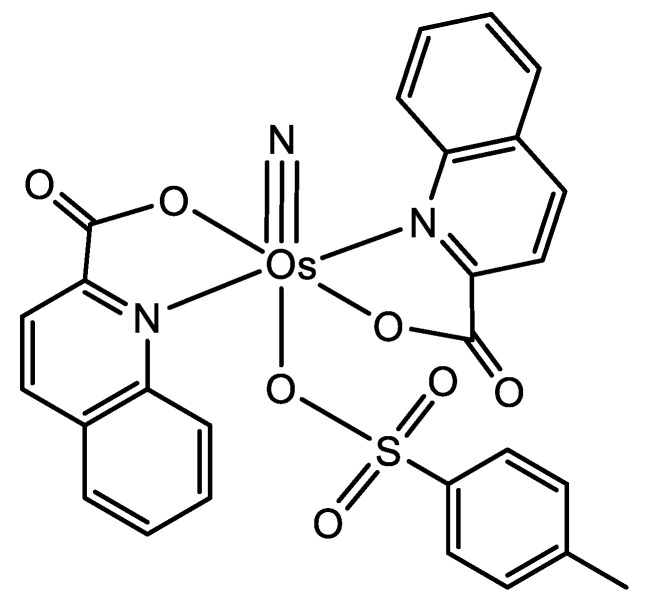
Structure of the osmium(VI) nitride complex. Adapted from [25].

In the same line of work, Kwong et al. reported [25] the application of the osmium(VI) nitride complex (Figure 5) for alkylbenzene oxidation in H_2_O_2_ and obtained similar results. The reaction was performed at a lower temperature, 23 °C, with 62.5 mM of H_2_O_2_ (aq. 30%, solution), 0.625 mM of the catalyst, and 1.25 M of *p-*xylene in a mixture of CH_2_Cl_2_/CH_3_CO_2_H (5:2 *v*/*v*). After 25 min, a 98% yield was obtained, based on H_2_O_2_, where the main products corresponded to phenols with 97% selectivity. This occurred even though the aromatic C-H bond had a higher bond dissociation energy (112 kcal mol^−1^), compared to the benzylic C-H bond (95 kcal mol^−1^). Other solvents were tested, and a major conclusion was that acetic acid’s presence enhanced the products’ yields, as happens in the AMOCO process. The experimental work was enhanced by density functional theory (DFT) calculations, the results of which are discussed in Section 3.

**Figure 6:**
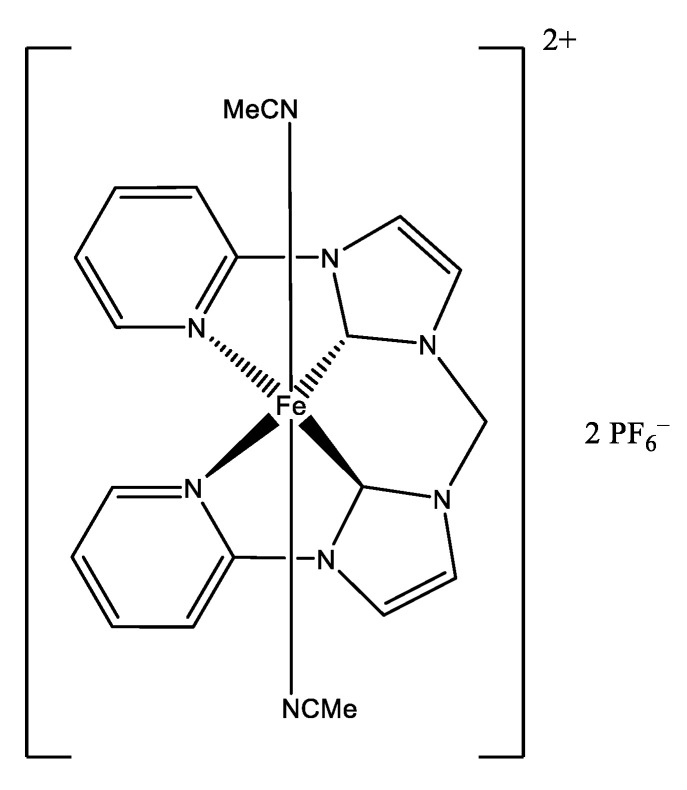
Structure of an iron(II) complex bearing a tetradentate bis(pyridyl-*N-*heterocyclic carbene) ligand. Adapted from [26].

Lindhorst et al. reported, in 2017 [26], a molecular iron-NHC complex (Figure 6) capable of catalyzing the oxidation of aromatic hydrocarbons including *p-*xylene. The authors were focused on the oxidation of the aromatic ring. Upon applying the optimized conditions (1 mol% catalyst vs. the substrate in acetonitrile, 0.25 equivalent relative to the substrate of H_2_O_2_ aq. 50%, at −10 °C for 1 h), the total conversion obtained was 12.6%, with phenol selectivity reaching 84.9%. Lower temperatures appeared to enhance the catalyst’s stability (9 cycles at 20 °C vs. 13 cycles at −10 °C), whereas a high concentration of oxidant significantly decreased the lifetime of the catalyst, and 2,5-DMP and 2,4-DMP were formed in the same order of magnitude.

**Scheme 3:**
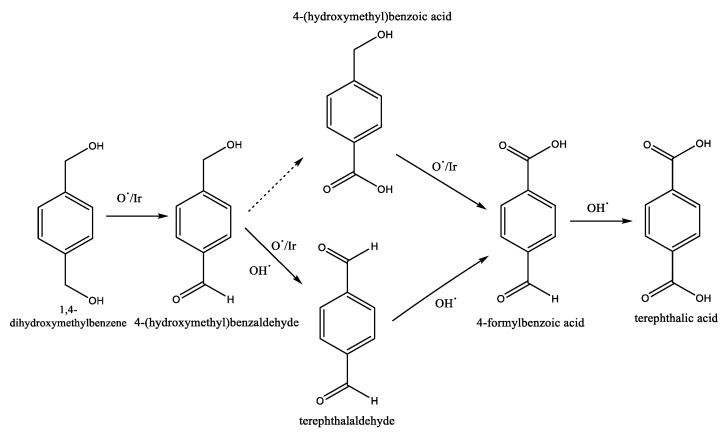
Proposed mechanism for the oxidation of 1,4-dihydroxymethylbenzene. Adapted from [27].

Goulas et al. [27] screened the oxidation of di-hydroxymethyl benzene (DHMB) to terephthalic acid using several metals supported on carbon, such as Au, Pt, Pd, Cu, and Ir. In the first test, performed at 90 *°*C and 1 bar of oxygen, Pd led to the highest conversion but low yields for the oxidation products. The same trend occurred with Pt. Both were reported to be good C-C bond scission catalysts. Metals are known for their oxidative power; for exampke, gold and copper only oxidized DHMB to HMBA, while the Ir catalyst obtained a 93% oxidation product yield, including 55% for TPA at full conversion of DHMB. When comparing all the metals at similar conversion of between 60 and 75%, Ir presented a minimal loss of carbon. Under optimized conditions, the Ir/C catalyst promoted the full conversion of DHMB, leading to a maximum yield of TPA of 76% after 20 h, at 100 °C, with 12 bar of O_2_. Under the same conditions, *p-*xylene did not react; therefore, this type of catalyst appears to be more suitable for a biomass-based processes, or later, oxidation within the full process. The authors explored the possible mechanism (Scheme 3) followed by the iridium catalyst. By subjecting the reactants and intermediate oxidation products to reaction conditions in the presence and absence of a catalyst, it was interestingly observed that DHMB was not converted in the absence of a catalyst, while HMBA and terephthalaldehyde were. The authors explained that the presence of a single aldehyde group activated the molecule for the radicalar reaction. Lastly, DHMB and 4-hydroxymethylbenzoic acid were inert in the absence of the iridium catalyst, reinforcing the radical hypothesis.

In the same year, Pan et al. [28] studied the catalytic oxidation of *p-*xylene to TPA by ozone in the presence of a cobalt catalyst. First, different salts of cobalt were tested under the following conditions: 110 °C, 0.10 mol of catalyst and 0.545 mmol of KBr per mol of *p-*xylene, 15 mg/L of ozone, a gas flow rate of 0.8 L/min, a duration of 6 h, and glacial acetic acid as a solvent. Cobalt chloride hexahydrate achieved conversion of ca. 50%, which was increased up to 70% when cobalt acetate tetrahydrate was used. The best result from the salts was obtained when cobalt acetate was used, allowing for almost full conversion (97%) of *p*-xylene with high selectivity for TPA (82%); this shows that the anhydrous reactional medium is mandatory for the success of the reaction. The increase in the concentration of ozone in the reaction medium did not affect the selectivity distribution of the products. The conversion of *p*-xylene, on the other hand, increased linearly with the increase in ozone concentration. After further optimization, such as altered catalyst and ozone concentration and temperature, the best conditions attained were: 80 *°*C, 0.10 mol catalyst per mol of *p-*xylene, an ozone concentration of 63 mg/L, a gas flow rate of 0.8 L/min, a duration of 6 h, and glacial acetic acid 17 mol/*p*-xylene mol used as the solvent. A total of 76% of *p*-xylene conversion was attained with 84% selectivity for TPA, which could be increased up to almost full conversion (96%) by adding KBr.

**Figure 7:**
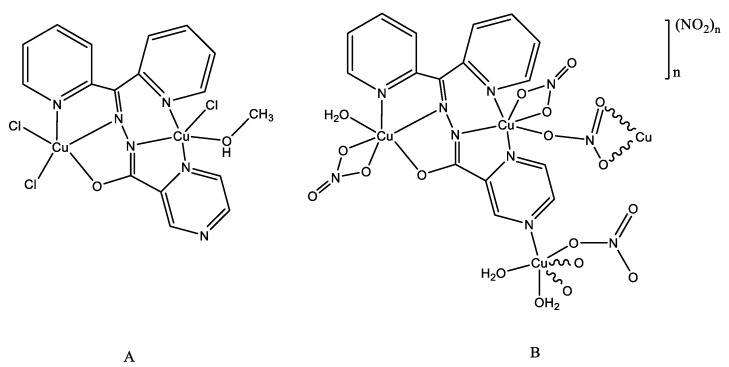
Structure of the copper(II) complexes derived from aroylhydrazone N’-(di(pyridin-2-yl)methylene)pyrazine-2-carbohydrazide; (**A**) Complex [Cu_2_(μ-1κN^3^,2κN^2^O-L)(Cl)_3_(MeOH)] (**B**) 1D polymer [Cu_3_(μ_3_-1κN^3^,2κN^2^O,3κNL)(μ-NO_3_)(NO_3_)_3_(H_2_O)_3_]n·nNO_3_. Adapted from [29].

New polynuclear Cu(II) complexes derived from aroylhydrazone N’-(di(pyridin-2-yl)methylene)pyrazine-2-carbohydrazide were reported by Sutradhar et al. in 2019 [29] as catalysts for the oxidation of *p*-xylene under mild conditions. Upon appling microwave irradiation (5 W, 3 h) or conventional heating (6 h), acetonitrile as the solvent, H_2_O_2_ (aq. 30%, 2:1 oxidant: substrate), and 2% mol of catalyst based on the substrate, methyl benzyl alcohol and *p*-tolualdehyde were the main products. However, no subsequent oxygenated products, such as 4-CBA or terephthalic acid, were detected. Under conventional heating and using the complex [Cu_2_(μ-1κN^3^,2κN^2^O-L)(Cl)_3_(MeOH)] (Figure 7A), the authors obtained a 32.8% total yield with selectivity for *p-*toluic acid of 38%, corresponding to the highest yield, 12.4%. Under microwave irradiation, the 1D polymer [Cu_3_(μ_3_-1κN^3^,2κN^2^O,3κNL)(μ-NO_3_)(NO_3_)_3_(H_2_O)_3_]n·nNO_3_ (Figure 7B) tended to favor the formation of methyl benzyl alcohol (39.7% total yield with 23.9% yield of methyl benzyl alcohol).

**Figure 8:**
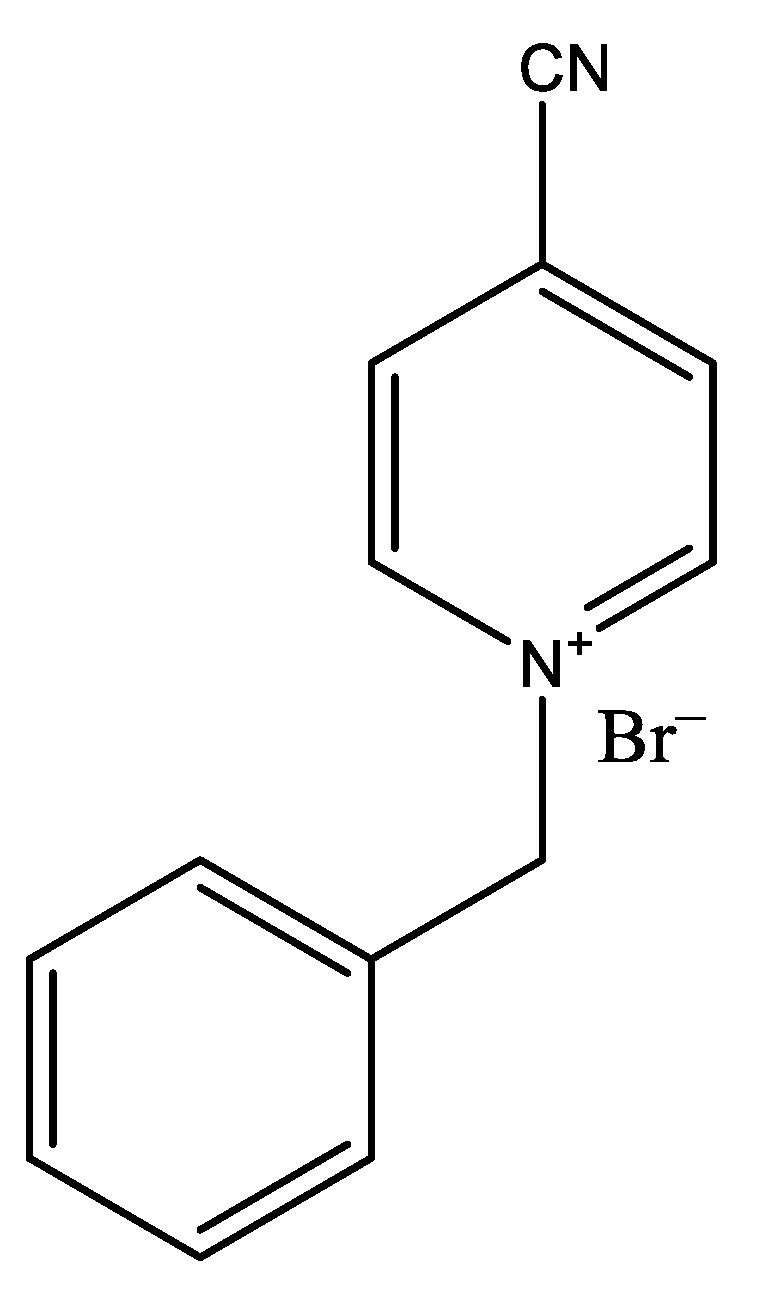
Structure of the 1-benzyl-4-*N,N*-dimethylaminopyridinium salt. Adapted from [30].

Zhang et al., in 2019 [30], screened the metal-free oxidation of *p-*xylene to *p-*toluic acid using *N*-alkyl pyridinium salts, expecting the use of mild conditions and better interaction with the organic substrate, using only a pure organic catalyst and avoiding over-oxidation caused by the metal-species. The general conditions used were: 0.5 mmol of 1-benzyl-4-*N,N*-dimethylaminopyridinium salt (Figure 8) as a catalyst for 10 mmol of *p-*xylene heated at 160 °C, an O_2_ pressure of 1.5 MPa, and a duration of 2 h. Before the reaction, 0.2 mmol of *p-*tolualdehyde was introduced as an initiator to reduce the induction period. The solvent-free reaction produced satisfactory results, with a maximum conversion of 52% and 86% selectivity towards *p*-toluic acid. The low conversion of xylene was probably due to the low solubility of the products and can be improved with the addition of solvent. While testing several solvents, the choice between acetic acid, acetonitrile, and DMF was clear. The highest conversion (88%) obtained was with acetonitrile, maintaining selectivity for *p-*toluic acid of 83%. While in the original paper, the authors compared their results to some metal catalytic systems that did not achieve great results, as seen in this review, other metallic catalytic systems can achieve similar results when lower temperatures or shorter times are applied (see Table 1)

**Scheme 4:**
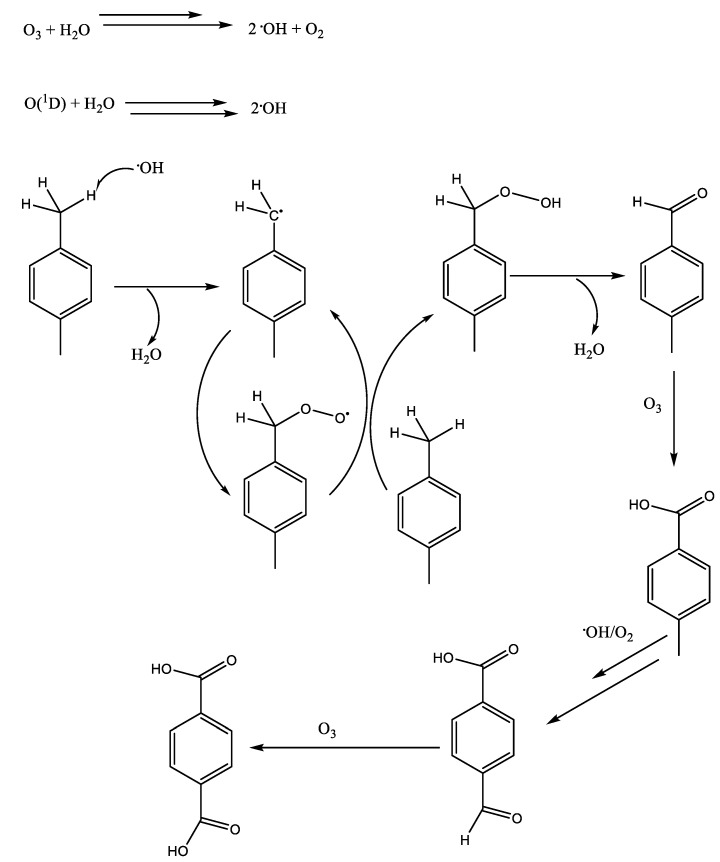
Reaction mechanism proposed by Hwang et al. for *p-*xylene oxidation in CH_3_CN-H_2_O with ozone upon UV light irradiation. Adapted from [31].

Another example of the use of ozone for the conversion of *p-*xylene to terephthalic acid was reported by Hwang et al. in 2019 [31]. This work presents an alternative to the AMOCO process with low energy demand and a greener process, avoiding the formation of CO_2_ and CH_3_Br. Using a 100 W Hg lamp (200 mW cm^−2^ at 310 nm) and a mixture of *p-*xylene:acetonitrile:water = 1:3:2 (pH = 4.5), and O_2_ gas, with ~10% ozone at room temperature, it achieved outstanding conversion of 98% with a yield of TPA of 96%. In the end, the authors reported an E-factor ($\frac{Total\;mass\;of\;waste\;from\;the\;process\;}{Total\;mass\;of\;product}$) of 0.118, while that of the AMOCO process varies between 3.14 and 10.14, with similar atom economy, carbon efficiency, and selectivity for TPA. The proposed mechanism, presented in Scheme 4, illustrates first the formation of the hydroxyl radical resulting from the interaction of ozone and water. The presence of water plays an important role at the start of the reaction. After that, the radical abstracts the proton from the methyl group of *p*-xylene, initiating the radical reaction until the last product, TPA, is obtained.

**Scheme 5:**
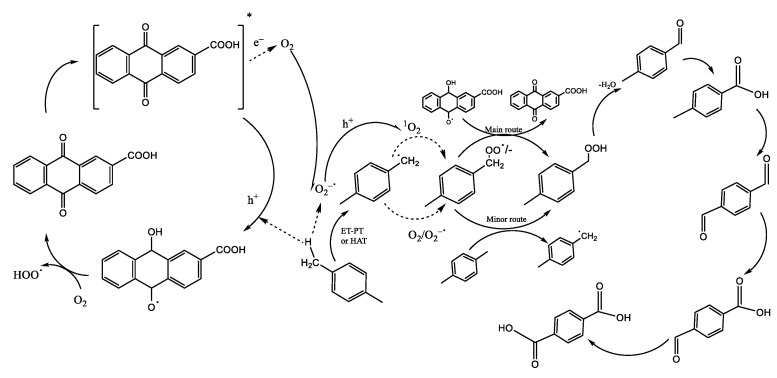
Mechanism of *p-*xylene photooxidation catalyzed by anthraquinone derivative with O_2_ under visible light. Adapted from [32].

Jiang et al. [32] explored the photo-catalytic oxidation of *p-*xylene using substituted anthraquinones as catalysts. 2-carboxyanthraquinone was the best catalyst in the study, being active under visible light (35 W tungsten-bromine lamp) and with 1 atm of O_2_ at r.t., achieving 70.9% conversion and 88.2% selectivity for *p-*toluic acid after 12 h of reaction. When the reaction was performed under air instead of O_2_, a small decrease in conversion and selectivity to 65 and 67%, respectively, was observed. One interesting result was the addition of benzenesulfonic acid, which not only increased the conversion of *p-*xylene to 86.7%, but also allowed the reaction to progress, achieving 27.2% terephthalic acid. Increasing the temperature to 50 *°*C reduced activity not only in conversion, but also in selectivity for TPA, resulting in a decrease in the stability of the excited state of 2-carboxyanthraquinone. The mechanism presented for this catalyst (Scheme 5) combined previously obtained results, as well as those from the literature and EPR spectra, showing that is possible to excite anthraquinones with visible light. When this process occurs, an excited state is generated where photoelectrons can be rapidly transferred to oxygen to form radical anions.

Table 1 summarizes some of the reaction conditions, as well as the main products and yields for the best systems reported with homogeneous catalysts.

### 2.2. Heterogeneous Catalysts

The field of heterogeneous catalysis provides new information on and highlights systems that can be efficient for the oxidation of *p*-xylene; for more information about this topic and to see previous results, [1,14,19,33] are good examples.

Qin et al. [34] focused on the production of terephthalaldehyde, a promising material for the pharmaceutical industry, among others. To achieve a safer process than the one currently in place, the authors reported the use of a fixed-bed reactor where 0.5 g of catalyst Fe-Mo-W was added, and then, *p-*xylene was vaporized at 0.01 mL/min at 300 °C. The oxidation of *p-*xylene occurred using air as an oxidant, with a liquid space velocity of 1.2 mL gcat^−1^ h^−1^ at 500 °C. During the optimization process of the multiple parameters, some important conclusions could be drawn. For example, at the optimal temperature, the variations in enthalpy and Gibbs free energy of the reaction in the gas phase were less than zero. In addition, the reaction was highly exothermic meaning, which is favored by low temperatures. So, a compromise must be reached to activate the molecules of *p-*xylene to react and avoid decomposition into CO_2_ and water. In the end, the Fe-Mo-W calcined at 550 *°*C achieved a conversion of *p-*xylene of 74% with a 73.6% yield of terephthalaldehyde and stability of about 50 h.

Lee and co-workers reported [35] the use of supercritical carbon dioxide for the synthesis of terephthalic acid via the partial oxidation of *p-*xylene in the presence of CoBr_2_. The authors screened the reaction for temperatures between 50 and 165 *°*C and for a maximum of 24 h of reaction time and, like previous authors, a large portion of *p-*xylene was lost during the process, probably through conversion into CO_2_. In this case, CO_2_ was already used in excess; thus, it was not possible to quantify the loss. The best result reported in this work achieved a terephthalic acid yield of 33.7% after 24 h at 150 *°*C and 17 MPa using supercritical CO_2_ and CoBr_2_. It is a longer process compared to other supercritical processes, such as the one by Dunn et al., which, at 280 *°*C and 24 MPa, in the presence of supercritical water and MnBr_2_, after 7.5 min, produced a TPA yield of 57%. The poor results obtained could be mainly due to the insolubility of CoBr_2_ in scCO_2_. It could be seen that as the catalyst load increased (due to the higher surface area available), the yield of terephthalic acid increased. Additionally, when compared with other cobalt catalysts such as cobalt(II) stearate and cobalt(II) hexafluoroacetylacetonate, this exhibited a higher yield in the same conditions as CoBr_2_ due to being homogeneous in the system. Another important factor studied is the influence of acetic acid or water in the reaction medium, and just a small amount of 1 mL of additive/mL of PX increased the yield obtained from 8.4% to 29.7% for acetic acid, and 23.7% for water. This increase could be explained by the increase in the homogeneity of the system, where PX and the catalyst are now more dissolved on scCO_2_. While this system did not improve the previously obtained results, it gives some insight into how to progress in the field of the supercritical catalysis of *p-*xylene and what to consider when projecting the experiments.

Li et al. prepared a metal-doped mesoporous material, Cu-MCM-41, to be used as a catalyst to produce 2,5-dihydroxyterephthalic acid in a one-step reaction starting from *p-*xylene. 2,5-Dihydroxyterephthalic acid is a derivative of the terephthalic acid used as an intermediate in the pharmaceutical industry and in batteries, and a linker to the synthesis of MOFs. [36,37,38,39,40,41] The synthesis of this compound usually requires harsh conditions such as high temperatures and pressures, multiple steps, and non-environmentally and health-friendly reactants; therefore, the search for greener and softer conditions is intense. By using the mesoporous material MCM-41, dopped with copper on a scale of Cu:Si = 1:100, it was possible to achieve a moderate conversion of *p-*xylene of 21.7% with relatively high selectivity of 73% for the product in question after 5 h of reaction at 80 *°*C, using 30% H_2_O_2_ as an oxidant, and with acetonitrile and acetic acid in a volume:volume proportion of 7:3 [42].

**Figure 9:**
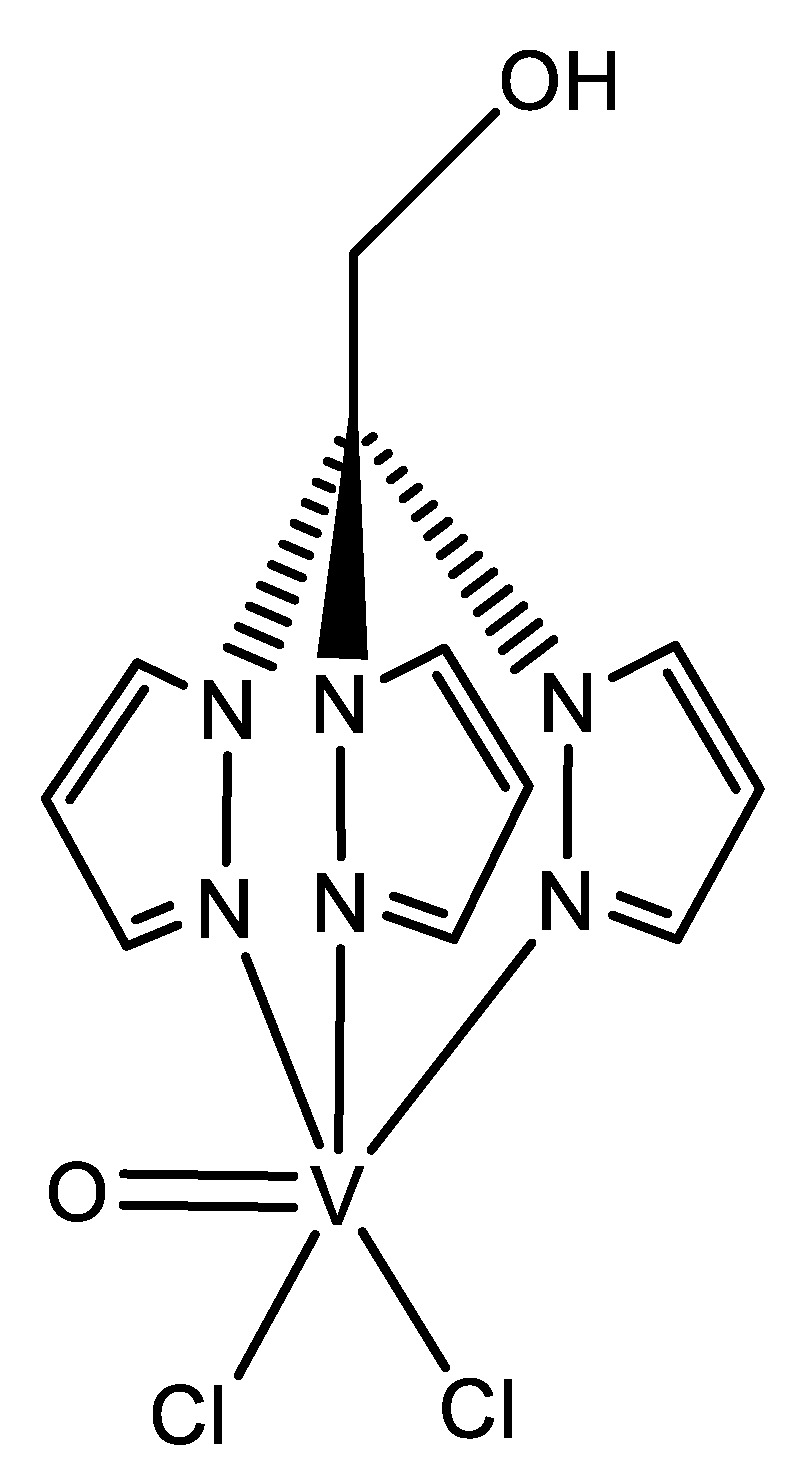
Structure of the vanadium(IV) C-scorpionate complex. Adapted from [43].

The use of C-scorpionate complexes as catalysts for xylene oxidation was also explored in 2017 by Wang et al., [43] where vanadium(IV) C-scorpionate complexes (Figure 9) were supported in functionalized carbon nanotubes and tested as catalysts for this reaction. In this work, again, the synergy of microwave irradiation with the reaction was remarkable. Under the same conditions, it was possible to obtain a 31% yield of p-toluic acid using microwave irradiation, where conventional heating led to 7.5%, showing that microwave irradiation provides very fast initial heating, enhancing the reaction rates. As proven before for C-scorpionate catalysts, the addition of an acid addictive such as nitric acid promoted the reaction. The immobilized catalysts were proven to be stable for up to six catalytic cycles, and in the best conditions (5 h of microwave irradiation, 80 °C, solvent-free, TBHP 70%, an oxidant:substrate ratio of 2:1, 3.2 × 10^−2^ mol% vs. the substrate, and nitric acid (10:1 additive:catalyst)), they obtained a yield of 43% p-toluic acid. Since it is a solvent and a KBr-free system, this already presents some advantages.

Samanta and Srivastava [44] explored the use of an FeVO_4_ graphitic carbon nitride (g-C_3_N_4_) nanocomposite for the oxidation of various cycloalkanes, including *p-*xylene. In their work, they compared catalysts using conventional heating (catalyst (50 mg), 60 °C, 4 h, H_2_O_2_:substrate = 2.5) and photocatalysis (catalyst (50 mg), 20–25 °C, 4 h, H_2_O_2_:substrate = 2.5, 250 W high-pressure visible lamp > 420 nm). Overall, the nanocomposite with a FeVO_4_:g-C_3_N_4_ wt% of 37 was found to be more active in the conditions of the study, and for *p-*xylene oxidation, achieved a yield of 21.3% for 4-methyl benzaldehyde in conventional heating and 34.4% for the photocatalytic system. Although the photocatalytic system presents an improvement, it is not as remarkable as for the other substrates, where the difference between the two systems is great, achieving double or even triple the yield.

Nicolae et al. reported [45] manganese iron oxides (Mn/Fe/O) as heterogeneous catalysts using hydrothermal treatment (HT) and citrate methods (CIT), and applied them to the oxidation of *p-*xylene using green conditions. The choice of oxidant was important. Upon comparing the activity of both catalysts, Mn/Fe/O_HT and Mn/Fe/O_CIT, with the different oxidants, it was observed that both catalysts were inactive when molecular oxygen or H_2_O_2_ were used, with a maximum conversion of 4%. On the other hand, when TBHP was used, the conversion exponentially increased to 85% and 77% for Mn/Fe/O_CIT and Mn/Fe/O_HT, respectively. This phenomenon was explained by the authors as TBHP being more stable than H_2_O_2_ and producing radical species that were more stable, as well, for molecular oxygen is known to have a lower oxidizing characteristic, often needing more temperature to become reactive and being a very stable molecule. The quantity of the oxidant is also an important factor; as stated before, some catalytic systems are influenced by the presence of water, and in this case, the increase in the oxidant did not increase conversion or selectivity for the last product of the oxidant when it was screened from a ratio of 1:4 to 1:12. When the effect of temperature was evaluated, it showed that below 80 *°*C, there was a significant decrease from 85 to 57% when Mn/Fe/O_CIT was used, and for temperatures above 80 *°*C, the reaction progressed, forming 4-CBA. Although the authors consider this as a negative point, with the focus being on *p-*toluic acid, it could indicate that this catalyst could actively produce TPA. Lastly, the recyclability of the catalyst showed a loss of activity of around 15% between the first and second cycles, but no further investigation was conducted; it could have been interesting to see if the catalyst continued to lose activity or if it would stabilize at one point, and to determine how many cycles it would take. In the end, under the best conditions (2.5 mmol of PX, 50 mg of catalyst, 2 mL of acetonitrile, *p*-xylene/TBHP = 1:4, 24 h, 100 *°*C) Mn/Fe/O_CIT was shown to be the more active catalyst, converting 98% of the substrate with a selectivity of 95% for *p-*toluic acid.

Wang and co-workers [46] reported the oxidation of *p-*xylene, this time to 4-hydroxymethylbenzoic acid (4-HMBA), under mild conditions using Cu-MOF as catalysts. Previously the same authors reported the use of Cu-MCM-41 as a catalyst to produce 2,5-dihydroxyterephthalic acid in a one-step reaction. This time the reaction took place at 30 °C using 30%H_2_O_2_ as an oxidant, and in the first optimization of temperature with this catalyst, it was observed that higher temperatures did not favor the conversion or selectivity of the reaction. Copper ions were detected in the liquid phase at high temperatures, indicating dissociation of the MOF, leading to the over-oxidation of 4-HMBA and to the decomposition of H_2_O_2_. At a lower temperature, selectivity for 4-HMBA reached 99.3% with a *p-*xylene conversion of 28.6%. After the optimization of the amounts of oxidant and solvent, and of the duration of the reaction, the authors reported conversion of 85.5% with high selectivity of 99.2% for 4-HMBA using acetonitrile as the solvent at 30 *°*C, for 5 h, with 30 mg of Cu-MOF, and with an oxidant:substrate ratio of 5.8:8.1 mmol; not only did it achieve incredible results at low temperatures and reaction times, but the catalyst maintained its activity for five cycles without losing any activity.

In 2019, Karakhanov et al. [47] doped a hierarchical mesoporous MCM-41/halloysite nanotube composite with the bimetallic MnCo catalyst (ratio 1:10) and screened its activity for the oxidation of p-xylene to TPA under the conditions of the AMOCO process. Therefore, the authors applied the new supported catalyst using acetic acid as a solvent, in the presence of KBr and with molecular O_2_ (20 atm) as an oxidant, for 3 h, at 200 °C; they achieved almost full conversion of *p*-xylene, with the main product being the desirable TPA at over 95% selectivity. When comparing the homogeneous system (using only the acetate salts of the metals in the same proportion) to the heterogenous one tested here, TOF increased exponentially from 37 to 142 h^−1^. The authors also provided an insight into the reaction conditions’ importance in the process. For example, a decrease in the temperature to 150 °C led to a significant decrease in the conversion (99% to 37%) and, more importantly, in the yield of TPA, which, at lower temperatures, did not achieve 1%. The influence of O_2_ pressure and the presence of KBr were also studied. The oxygen pressure is relevant to the TPA yield, whereas the presence of KBr is important to the overall reaction (a decrease to 2.2% of p-xylene conversion in its absence was observed). Although the catalyst could look promising for industrial processes, it exhibited a serious problem of metal leaching. After the first cycle, the cobalt content decreased from 1.29%w/w to 0.22%w/w in the second cycle, while manganese, which was already present in a small proportion, was leached after the second cycle from 0.15% to 0.03%. The authors proposed that leaching occurred due to the dissolution of metals by the solvent and hydrobromic acids in the reaction medium.

**Scheme 6:**
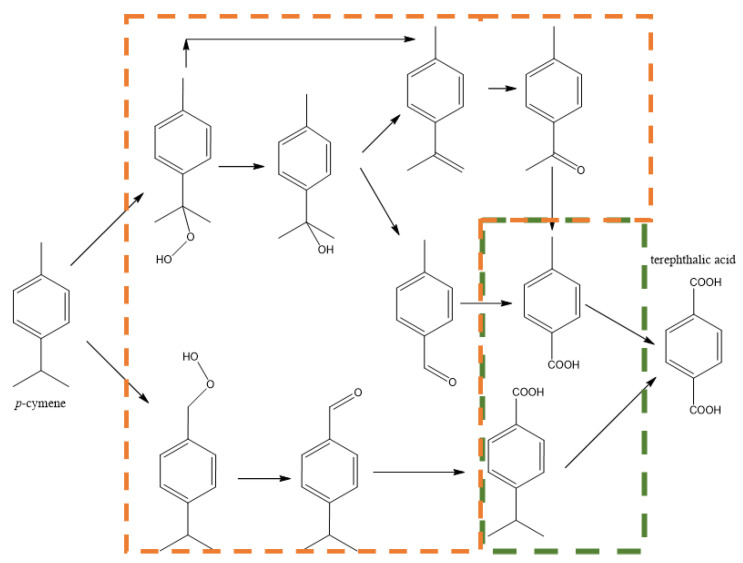
Reaction pathway for the oxidation of *p-*cymene, with low oxidation products in the orange box and high oxidation products in the green box. Adapted from [52].

Trandafir et al. disclosed an alternative and renewable raw material to produce terephthalic acid. In their study, manganese–cobalt mixed oxide catalysts, prepared using the co-precipitation and citrate methods, were applied as catalysts for the selective liquid phase oxidation of *p-*cymene to TPA (Scheme 6). While the oxidation of *p-*xylene to TPA is very straightforward, starting from *p*-cymene is more complex and consists of parallel and consecutive reactions. These can be divided into two parts (low oxidation products and advanced oxidation products) and the last product, TPA. All catalysts in this study produced full conversion of *p-*cymene but with different distributions of products. The catalysts prepared via co-precipitation presented the overall best distribution, with higher amounts of the advanced oxidation products and TPA when compared to those of the citrate method. The authors refer to the acidity of the catalyst as an important factor in an autoxidation mechanism. Studies on the autoxidation of p-xylene to benzaldehyde and the oxidation of cyclohexanone to adipic acid with Mn/Co catalysts have proven that better activity is linked to stronger acidity [48,49,50,51]. The best result was obtained by 1Mn2Co_pp, referring to the catalyst prepared via co-precipitation with a metal ratio Mn: Co of 1: 2, with an advanced oxidation product yield of 67% with a 10% yield of TPA (reaction performed at 140 °C, 20 atm of O_2_, no solvent, O_2_/*p-*cymene = 6/1, 2 mmol of *p-*cymene, 16.7 mg of catalyst). Finally, when the stability of the catalyst was tested for three consecutive runs, the authors observed a slight decrease in *p-*cymene conversion, a total of 9%, with selectivity for the main products remaining constant [52].

**Scheme 7:**
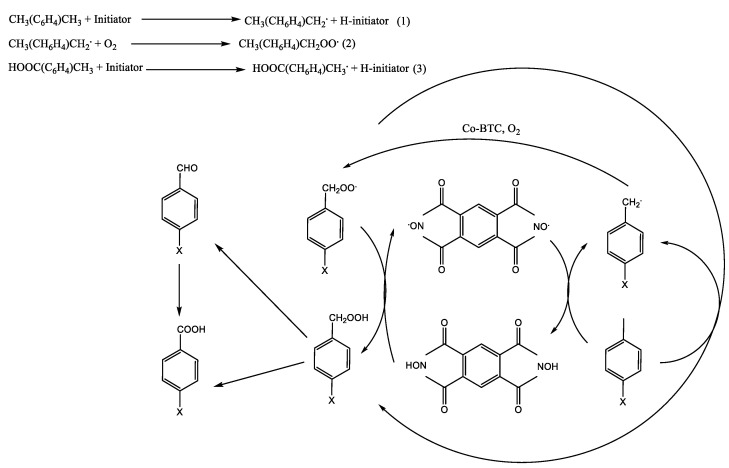
The mechanism proposed for the oxidation of *p-*xylene, which is catalyzed by Co-BTC/NDHPI X = CH_3_, COOH. Adapted from [53].

Xu et al. [53] tested the efficacy of the MOF cobalt–benzenetricarboxylate (BTC) when in conjunction with NHPI, since this compound is a very effective catalyst for oxidation under mild conditions and as an initiator. Both catalysts alone could not perform the oxidation of *p-*xylene at 100 °C, and after 12 h, no product was detected (using acetonitrile as the solvent and 3 MPa of O_2_); however, when used in combination, the conversion of *p-*xylene reached 57%, with the main product being *p-*toluic acid. By increasing the reaction temperature up to 150 °C, the previous system with only NHPI started to achieve 93.5% conversion, with TPA selectivity of 67.5%, while the Co-BTC could not achieve conversion higher than 3%. Again, when both catalysts were in use in the same reaction, the system achieved full conversion of *p-*xylene, and the TPA selectivity increased from 67.5 to 96.2%. Finally, the stability of Co-BTC was tested, and after the reaction, the MOF was separated, washed, dried, and applied in a new cycle. After two cycles, the Co-BTC maintained the same activity as before, and SEM images showed the catalyst remained nearly as spherical as it had previously. The proposed mechanism, presented in Scheme 7, showcases, in the first equation, the common oxidation of hydrocarbons. PX reacts to form a *p-*methyl benzyl radical, which has strong resonance stabilization (Equation (2)). As stated before, *p*-toluic acid is even more difficult to oxidize due to the presence of the electron-withdrawal group, but the presence of NHPI as an initiator and of the catalyst allowed this difficulty to be overcome.

In one of the most recent publications about this theme in 2022, Wang et al. [54] applied α-Al_2_O_3_-supported VMOx (M = Ag, Mn, Fe, Co, etc.) as a catalyst for the oxidation of *p-*xylene. The catalysts were prepared via incipient wetness impregnation with a vanadium load of 5.4 wt% and tested under the same conditions (295 °C, 1 vol % *p-*xylene, *p-*xylene/O_2_/He = 1:3:96, V_total_ = 40 mL/min, GHSV = 24,000. From all the metals, the VAgO_x_ mixture presented the best reaction rate (82.3 mg^−1^ g^−1^ h^−1^) and selectivity towards *p-*methyl benzaldehyde, and the optimum nAg/nV ratio was located at ca. 0.4–0.5.

Table 2 summarizes some of the best reaction conditions, as well as the main products and yields for the different systems reported, with heterogenous catalysts for PX oxidation.

The oxidation of alkylarenes is an extensive and complex topic and can sometimes produce some important information on the oxidation of *p*-xylene, since it is one of the many substrates tested. Although the majority of results and studies focus on the production of alcohols and aldehydes, we wanted to highlight a few interesting results that could represent important advances in the production of TPA.

In 2019 Sarma el al. [55] tested zinc oxide loaded with copper as a photocatalyst at room temperature and in the presence of oxygen. When *p*-xylene was tested, the product obtained was terephthalaldehyde (the two methyl groups converted to aldehyde simultaneously). Additionally, 4-methyl benzyl alcohol and p-tolualdehyde were tested, and the catalyst was able to convert the methyl group to aldehyde, with the lowest yield being 66% and the highest 82% in a maximum reaction of 24 h. This way, it is possible to avoid the formation of *p*-toluic acid by maintaining the most reactive group present (alcohol and benzaldehyde).

In a similar way, Dutta et. al. [56] reported, in 2020, that the use of a ruthenium(II) complex achieved interesting results at 60 °C in the presence of TBHP. While oxidizing 4-methyl benzyl alcohol, it was possible to oxidize the methyl group to an aldehyde (with a yield of 65% after 3 h), maintaining the alcohol in the aromatic ring and avoiding the formation of p-toluic acid. Similarly in the same study p-xylene was tested and both methyl groups were oxidized to the aldehydes (after 4 h, with a yield of 74%), again avoiding the formation of p-toluic acid.

Lastly, in 2022, Zhang et. al. [57] used Pd/C as a catalyst at 80 °C, dimethylacetamide, and a small amount of water and NaOH to perform selective oxidation of 4-methyl benzyl alcohol, achieving, after 48 h, a 4-hydroxybelzandehyde yield of 62%.

Overall, the study of alkylarenes is extensive, with several review articles [58,59,60,61,62] available to obtain a wider understanding of this topic, and could be an answer to improving the production of TPA.

### 2.3. Biocatalysis

The topic of biocatalysis is significant and extensive. It provides an alternative to other methods and helps to understand the mechanistic pathways involved in reactions [63,64].

Luo and Lee [65] approached the transformation of *p-*xylene into TPA in a different form. While most of the research is focused on metallic catalysts or changes in the setup, these authors decided to find a bio-approach to the TPA production problem. Several process developments related to biomass-derived *p-*xylene production allowed the authors to search for a microbial strain that could convert *p-*xylene into TPA, and it was found in an *Escherichia coli* species. The authors divided the system into two parts: first, the conversion of *p-*xylene into *p-*toluic acid, and then, to TPA. With some genetic modification and after optimization of the process, the authors reported a maximum production of TPA of 0.748 gL^−1^h^−1^, meaning 4.5 mmolL^−1^h^−1^.

## 3. Computational Studies and Simulations

**Figure 10:**
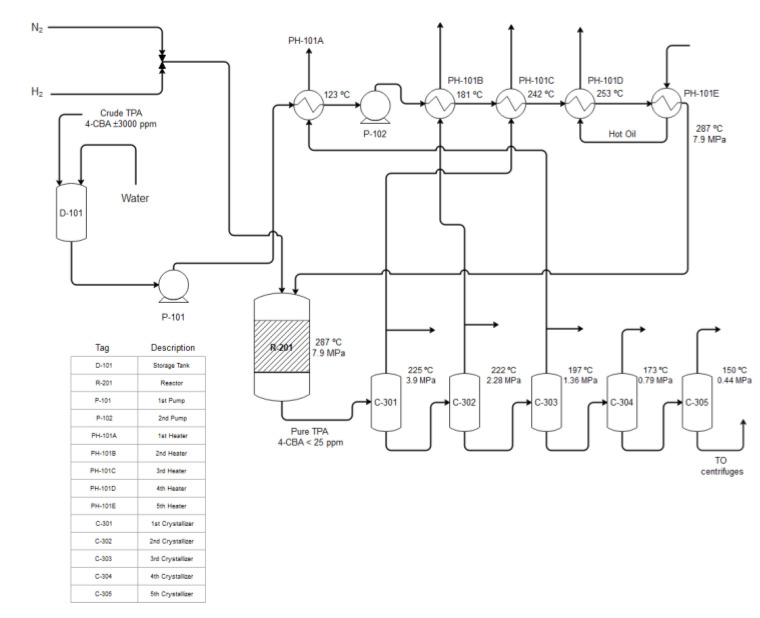
Flow sheet of an industrial hydropurification plant for TPA. Adapted from [68].

**Figure 11:**
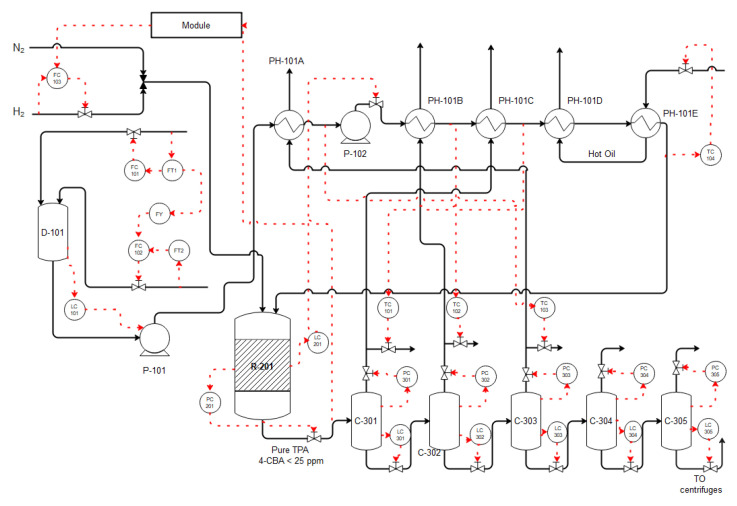
Flow sheet of the industrial hydropurification plant for TPA with improved PID control. Adapted from [68].

In 2016, Li et al. simulated and improved the dynamic model of a purification process (Figure 10) by applying the IFSH methodology, considering its characteristics and catalyst deactivation, with the inclusion of some energy integration. First, the authors simulated steady-state and dynamic models with kinetics adjustments resulting from previous work [66], showing that in the case of the steady state, these new kinetics parameters were fitting when compared to the real plant. The dynamic simulations used the real size of the plant and were also compared to the previous dynamic model by Azarpour and Zahedi [67], including the deactivation equation and values for the catalyst due to sintering. The total simulation time was 8000 h, but the real data are only available for the first 5000 h due to the shutdown of the plant. During that period, the simulation overlaps with the experimental data very fittingly. Some key parts of the simulation, such as the plant control objectives, were fixed, guaranteeing normal function of the unit (for example, avoiding cracking of the catalyst bed or maintaining the reactor temperature in the range of the reaction kinetics in consideration, and most importantly, keeping the content of 4-CBA below the 25 ppm). When analyzing the steady-state model, temperature exhibited a larger steady-state gain on the 4-CBA fraction, but due to the restriction of the reactor due to catalyst deactivation, the hydrogen variable was selected as the main variable. As an objective function, a profit equation considering the production volume, as well as material and energy costs, was put in place. After testing the influence of control and applying PID controllers, valves, and their gains and delays, including a module which functioned as quality control by receiving information on the exit concentration of 4-CBA, a performance assessment of the plant was conducted (Figure 11). It included the important parameters of the settling time, deviation from the production target, and final profit, and in the end, sixteen main control loops were involved in achieving a good control system that distinguished itself from the industrial one due to its quality controller for 4-CBA. Three types of disturbance were tested: the 4-CTA feed flow, the concentration of 4-CTA slurry, and 4-CTA flow with different impacts on the system. However, the difference before and after the implementation of the control system was evident; for example, a disturbance on the 4-CTA feed flow would deactivate the catalyst at 3630 and 4250 h (depending on the disturbance), while after the implementation, this only occurred at 5520 and 5890 h. This delayed effect is seen with the disturbance of other variables with different degrees of intensity, showing improvement in the system with increased profits, controlled purification of TPA, and an increased lifetime of the catalyst [68].

**Scheme 8:**
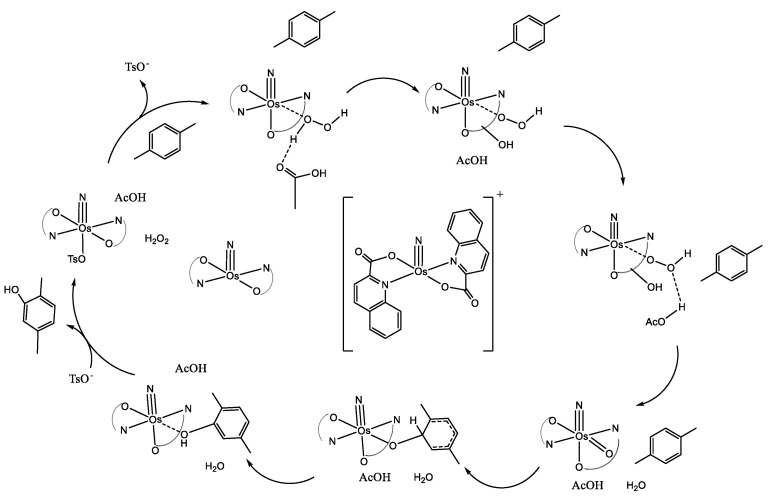
Reaction mechanism for the reaction of DFT-derived *p-*xylene. Adapted from [25].

As reported above, in 2017, Kwong et al. [25] studied the oxidation of *p-*xylene using an osmium(VI) catalyst, and to gain more insight into this particular catalytic system, DFT was studied (Scheme 8). The catalytic systems involved the binding of H_2_O_2_ to the Os^VI^ center, which, after a change in geometry, led to heterolytic O-O bond cleavage, leading to water formation and an Os^VIII^ nitride oxo species. This new species is highly reactive and, due to different attacks on *p-*xylene, led to different products. The attack of the ortho position led to the formation of 2,5-dimethylphenol, and it could also attack the ipso position, but the transition state was less stable (18.1 vs. 17.1 kcal/mol), which agrees with the selectivity trend obtained (85.3 vs. 11.9). The methyl attack was even less stable, with a barrier of 19.5 kcal/mol, which corroborates the results obtained experimentally. Interestingly, DFT calculations showed that side-chain hydroxylation can occur via hydride transfer, which directly forms a hydroxylated product without any rebound barrier.

Several authors report the liquid oxidation of *p-*xylene as a first-order reaction, but gas-phase oxygen follows zero-order kinetics. To study the influence of oxygen partial pressures on the liquid phase oxidation kinetics of *p-*xylene, Shang et al., in 2014, investigated reaction kinetics mimicking industrial conditions. Two sets of experiments were tested: a batch system and a continuous system. The kinetic mechanism for the oxidation of *p-*xylene involved Co^II^, Mn^II^, and Br^−^, so that when in acetic acid, the Co^II^ oxidized to Co^III^, later oxidizing Mn^II^, which, in the end, created a bromine free radical. The bromine free radical will abstract the alpha-H atom from the hydrogen-containing groups on the benzene ring, later making an alkyl free radical in addition to oxygen, resulting in the formation of an alkoxyl free radical. In previous works, this step was assumed to be instantaneous due to high oxygen partial pressure, but since this parameter was studied this time, it was considered a rate-determining step. The rest of the mechanism remains unchanged and derives from the other publications and examples demonstrated above. In the model equations, there exist 44 parameters to be estimated, and this high number can lead to difficulty and overfitting, making the estimated parameters unreliable. Therefore, it was necessary to reduce this number by simplifying some elementary steps, such as the reaction of ROO^−^ and ROCOOO^−^ for removing the alpha-H atom of a certain substrate, that were identical. Additionally, all of the substrates had equivalent approximated initiation rate constants, and the reaction rate constants of oxygen to different alkyl and acyl radicals were the same. Starting with the batch experiments, it was observed that when the oxygen volume fractions in the outlet were decreased to 3%, the kinetics obtained were very different from those when this value was higher, showing the importance and effect of oxygen partial pressure in the kinetics of the reaction. When the authors compared their models with others published with different orders, it was possible to see that those models could not accurately represent the kinetics at 3%, mainly due to not considering the importance and influence of oxygen partial pressure. To continue to verify the validation of the model, continuous experiments were then carried out in which the gaseous and liquid reactants were fed into the reactor continuously. The yield of TPA was calculated as a function of the oxygen volume fraction, and it was observed that the simulation could predict the results obtained in the experiments. Moreover, when the volume decreased from 5%, the yield of TPA started to reduce gradually and the model could represent that change. From this report, a kinetic model where the oxygen partial pressure is taken into consideration was created, and it was shown that there exists a threshold for this pressure that will influence *p-*xylene oxidation kinetics [69].

The same authors also later modulated CO_2_-assisted *p-*xylene oxidation using batch system experiments. The kinetic model was the same as the previous one, including the simplifications, except for the addition of CO_2_, which was proposed to form a peroxocarbonate that may participate in the formation of the free radical chain by oxidizing Co^II^ to Co^III^. From the experiments conducted, it was concluded that the presence of CO_2_ enhances the reaction, achieving the best results in a shorter time when compared to the reaction performed with air, but the range of temperatures studied did not influence the rate constants significantly. The last parameter was the quantity of the catalyst, and in this case, only the increase in HBr was proven to affect the rate constant, which doubled when double the quantity of HBr was used; this indicates that in the presence of CO_2_, the conversion reaction between Co(II)/Co(III) and Mn(II)/Mn(III), as well as the steps in the oxidation of HBr by Mn(III), are fast enough that the concentration of the bromide free radical is roughly equal to that of HBr. In the end, a kinetic model for CO_2_-assisted PX oxidation was developed, and it was capable of calculating the values of the reactants, intermediates, and desired products that matched the experimental data well [70].

## 4. Reactor and Purification Step

**Scheme 9:**
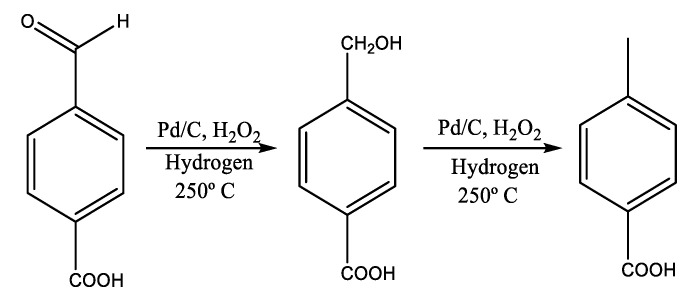
Reaction pathway and products obtained for the hydrogenation of 4-CBA.

**Scheme 10:**
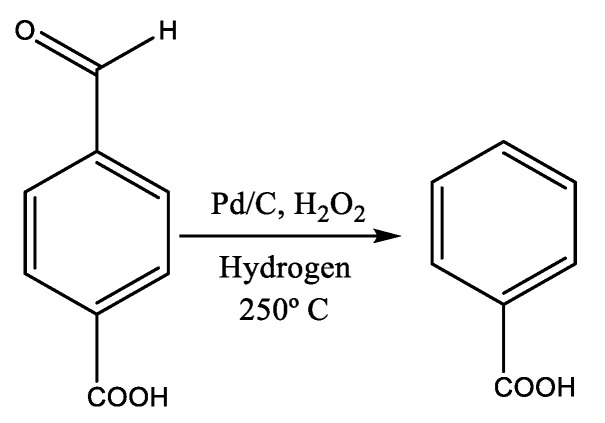
Reaction pathway and the product obtained for the decarbonylation of 4-CBA.

The purification of terephthalic acid is an important process in the production of this compound. As stated above, during the process of production, TPA is contaminated with 4-CBA at a mass fraction of around 3000 ppm, whereas commercial TPA must contain less than 25 ppm. To treat and reduce this quantity, crude TPA is sent after synthesis for hydropurification, a highly energy-consuming (high temperatures and pressure) and catalytically costly process (deactivation of the palladium catalyst due to Pd sintering, poisoning by sulfur and other elements, mechanical destruction, corrosion, and fouling) [67,71]. During this process, 4-CBA is transformed into TPA as described in Scheme 9, while a side reaction of decarbonylation to benzoic acid can also occur (Scheme 10) [12,72].

In 2019, while researching the oxidation of *p-*xylene to TPA, Hwang et al. [31] proposed a different purification process for the removal of *p-*toluic acid and 4-CBA from the TPA formed. In this case, the reaction taking place in the presence of ozone and a UV lamp would precipitate the TPA, since this is less soluble than the other two compounds in the *p-*xylene–acetonitrile–water (1:3:2) solution. The first precipitate formed during their study, which contained 32 mol% of *p-*toluic acid and 160 ppm of 4-CBA, was washed three times with an acetonitrile–water solution (3: 2, pH = 4.5), dissolving and removing the *p-*toluic acid and 4-CBA from the precipitate TPA. In the end, only 3 mol% of *p-*toluic acid was present and the 4-CBA decreased to 4 ppm (which is lower than the current industrial hydropurification treatment), without needing a high energy input or producing high volumes of wastewater, since the washed acetonitrile–water solution can be further exposed to the same conditions and convert the *p-*toluic acid and 4-CBA to precipitate TPA.

In 2020, Kuznetsova et al. [73] studied the effect of additives during an oxidation process performed to obtain a pure form of TPA with a low content of 4-CBA. During the AMOCO process, the TPA, which had low solubility in acetic acid, precipitated together with intermediates that also had low solubilities, such as *p-*TA and 4-CBA; this resulted in a crude TPA with around 2% contaminants that inhibit the polycondensation of TPA. Pure TPA should not have more than 100 ppm of 4-CBA, and to achieve this, the crude TPA was subjected to a hydrotreatment purification step at high temperatures and pressure, and with a palladium catalyst. The authors then tried to find an alternative to this two-step process by mimicking the reaction conditions during the AMOCO process, and in the second step, increasing the solubility of TPA in acetic acid by adding 1-butyl 3-methylimidazolium bromide (BMIM Br) or NH_4_OAc, thus making the intermediates easier to further oxidize to TPA. By adding BMIM Br or NH_4_OAc, the acidity of the medium decreased, meaning this step had to be performed at a higher temperature and catalyst concentration to compensate for this “acidic loss”. In the end, the authors reported the possibility of using either of these two compounds to purify TPA. While NH_4_OAc required a lower temperature, BMIM Br produced a purer final product with an increase of only 20 *°*C, but most importantly, both produced a pure TPA without any loss of the originally produced yield and without introducing an expensive catalyst such as palladium.

**Figure 12:**
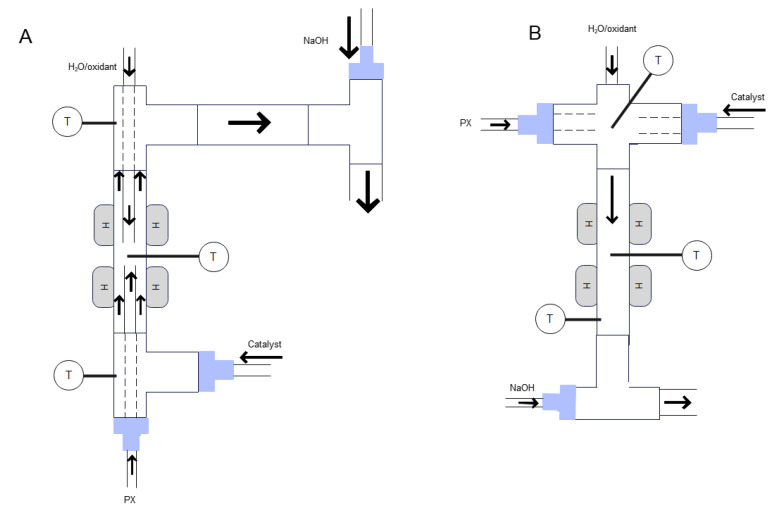
Representation of the (**A**) opposed-flow reactor and (**B**) tubular reactor, where elements labeled H are heating blocks. Adapted from [74].

Poliakoff and co-workers [74] studied the selective oxidation of *p-*xylene in sub- and supercritical water and the effects of geometry and mixing, since it was previously shown to be an important factor in the synthesis of metal oxide nanoparticles in supercritical water. The setup consisted of a coiled pre-heater at a supercritical temperature, which allowed for the total decomposition of H_2_O_2_ into a mixture of SC water and O_2_; CuBr_2_ and NH_4_Br as catalysts; and downstream of the reactor, NaOH, which neutralized CO_2_ caused by burning and prevented TPA from precipitating. In the study, two types of reactor were used: an opposed-flow reactor and a tubular reactor, depicted in Figure 12.

The opposed-flow reactor consists of a PX pipe that is concentric with the catalyst pipe, and both point upwards, where they meet the downward flowing stream of heated H_2_O + O_2_. All reactants should be efficiently mixed in the middle section of the reactor, after which they flow upwards to the outer section (concentric tube configuration) until the NaOH quench. In the tubular reactor, all reactants and solvents are mixed at the top of the reactor, which then lets the mixture flow downwards, where the NaOH quench solution is rapidly cooled at the bottom. The residence times for the opposed-flow reactor were not calculated accurately, but the authors considered that they should be around 2.3–3.3 s at 380 °C and 7.4–11.9 s at 330 °C, whereas in the tubular reactor, they were 5.8 s at 380 °C and 19.2 s at 330 °C.

The opposed-flow showed, at first, that at 330 °C, the extension of the reaction was very small, mainly due to the low activity of the catalyst. When the temperature was increased to 380 °C, the production of TPA increased, but an interesting result was obtained. Changing the retention time did not alter the yield obtained of TPA, which was constant even when it was decreased by a factor of 2. As explained by the authors, this suggests that most of the product is formed at the beginning of the reaction alongside the intermediates, which will later burn or decarboxylate. An important factor alongside geometry is the mixing of the reactants and the catalyst, for which it has been reported that efficient mixing tends to provide a high rate of reaction. Since the opposed-flow reactor already achieves efficient mixing and is not suited to alterations, a tubular reactor was used to test the importance of this parameter. Therefore, four different reactors, with different entrances of the reactants (without and with different T-pieces) and fed a biphasic mixture, were tested with different mixing efficiencies. The results confirmed the theory that the reactor where the mixing was the best achieved the highest selectivity of TPA when compared with the others (90% vs. 30%). Upon comparing the different reactors, the tubular with the best mixing achieved higher TPA selectivity with lower CO_2_ yield when compared with the opposed-flow reactor despite achieving similar selectivity, as a high increase in CO_2_ is associated with this type of reactor [74].

Subramaniam and his co-workers simulated a greener spray process to produce TPA and compared it to the conventional AMOCO process. Economic analysis and life cycle assessment were conducted, and comparisons drawn between the two processes. The AMOCO process is a well-known process and is described in the introduction of this review. The spray process simulated by the authors consisted of a spray reactor in which the liquid phase, containing dissolved PX, and the catalyst (the same as in the AMOCO process) in acetic acid were dispersed as fine droplets via a nozzle into a vapor phase containing O_2_. This reactor operates at 200 °C and 15 bar pressure, and the resulting stream is later sent to a three-stage crystallizer, a centrifuge, and a dryer, producing a high-purity TPA in one step (<25 ppm 4-CBA and <5 ppm TA) and avowing the need for a later purifying unit. The off-gas unit is similar to the one in the AMOCO process. The economic evaluation of both systems, including four variations of the spray process with various amounts of acetic acid in the feed, revealed (with the cost adjusted to June 2012) that in both processes, about 40% of the equipment costs are due to special equipment such as crystallizers, dryers, etc., but the estimated investment for the AMOCO process is around USD 302 million. When compared to the spray process cases, especially the two extremes where the quantity of acetic acid used in the feed is nearly 10 times more compared to the AMOCO and a similar amount at its lowest, an investment of USD 241 million and USD 136 million, respectively, would be required, representing 80% and 45% of the investment necessary to implement the AMOCO process. This difference is mainly due to the non-existent hydrogenation section. When production costs are compared, the spray process only achieves a difference of between 5 and 16% when compared to the AMOCO process, which lies within the typical range of uncertainty of such predictions, and is thus not a deciding factor in comparing both processes. The environmental analysis and simulations showed that when comparing the VOC emissions of a real BP plant with the emissions of this process, the latter produces half the emissions reported by BP. It is also important to note that the major contributor, which is not reported in the TRI data, is acetic acid, which represents 40 times more emissions than methanol. In terms of CO_2_ emissions, the spray process with the same acetic acid input emits only 23% compared to the AMOCO process, and this value increases up to 91% when the acetic acid input increases 10 times more. In the end, the overall spray process has been proven to provide both economic and environmental benefits when compared to the AMOCO process [75].

## 5. Challenges and Conclusions

For almost 10 years, several developments in the oxidation of *p*-xylene have been reported with catalytic systems, homogeneous or heterogeneous, that could change the industrial process and make it greener.

Homogeneous systems apply several times the catalysts and/or conditions of the AMOCO process, while other systems provide other changes such as the presence of metal catalysts, the absence of acetic acid, the application of green oxidants, or even the use of ozone. Typically, the systems are not strong enough to achieve full oxidation from *p-*xylene to TPA, and have toluic acid as a stop product.

Heterogeneous systems tend to necessitate a higher input of energy but can achieve a longer oxidation chain when starting with *p-*xylene, but they also have a high stopping point for toluic acid.

Overall, homogenous systems tend to be able to convert with less energy input but with no possibility of reusability, while heterogeneous systems can be more sustainable in terms of catalyst reuse. Nevertheless, most of the systems suffer from the same challenge: the oxidation of *p*-toluic acid.

One of the biggest challenges in the process of *p*-xylene oxidation to TPA (Scheme 1) is the activation of *p-*toluic acid. Not only does its low solubility in some solvents decrease the possibility of conversion into further oxidated products, but the electronic withdrawal effect on the aromatic ring also leads to high deactivation, making it hard to oxidize the second methyl group.

As depicted in Scheme 1, terephthalic acid is the sixth consecutive oxidation product of *p*-xylene. Thus, in designing new sustainable TPA production routes, the use of green peroxides (e.g., H_2_O_2_) as oxidant agents could require an initial high concentration of such species, which could lead to inhibition of the catalyst and/or to higher degradation of the oxidant, increasing the difficulty of obtaining the desired (TPA) product.

Finally, the terephthalic acid manufacturing process must be sustainable, environmentally friendly, and cheap, thus assuring the feasibility of the whole production and utilization chain on a large scale.

Thus, proper design of the catalytic process, including the catalyst itself, is crucial to accomplish the above aims while overcoming the highlighted issues. As reported in this work, huge developments in different synthetic strategies have been made in recent years, and further developments will come from the intense research activity devoted to reaching the sustainable oxidization of *p*-xylene to TPA.

## Figures and Tables

**Table 1:** Reaction conditions and best results for homogeneous catalysts focused on the oxidation of *p-*xylene or derivatives.

Authors	Reaction Conditions	Substrate	Main Product	Yield %	Ref
Wei et al.	110 °C, Co(II) aza-crowned dihydroxamic acid complexes, diglyme, O_2_, 5 h	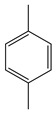	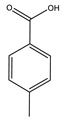	38 ^(a)^	[21]
Wang and Tong	Reflux, acetic acid, CoAc + MnAc + NHPI, O_2_, 14 h	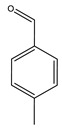	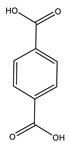	91 ^(b)^	[22]
Mendes et al.	35 °C, NCMe, H_2_O_2_, nitric acid (10:1 additive: catalyst), 5 min	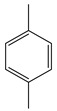	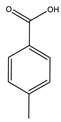	22 ^(c)^	[23]
Goulas et al.	100 °C, O_2_, Ir/C, 20 h	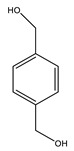	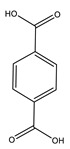	76 ^(c)^	[27]
Pan et al.	80 °C, CoAc, acetic acid, O_3_, 6 h	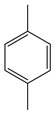	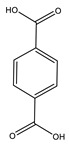	64 ^(a)^	[28]
Hwang et al.	r.t., NCMe + H_2_O, O_2_ + O_3_,	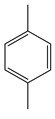	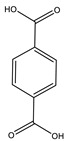	94 ^(a)^	[31]

^(a)^ HPLC yield; ^(b)^ isolated yield; ^(c)^ GC yield.

**Table 2:** Reaction conditions and best results for heterogeneous catalysts, focusing on the oxidation of *p-*xylene.

Authors	Reaction Conditions	Substrate	Main Product	Yield %	Ref
Wang et al.	80 °C, vanadium(IV) C-scorpionate@CNT, 5 h MW irradiation, solvent-free, TBHP 70%, nitric acid (10:1 additive: catalyst)	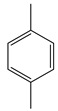	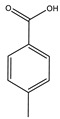	43 ^(a)^	[43]
Samanta and Srivastava	25 °C, FeVO_4_:g-C_3_N_4_, 4 h, H_2_O_2_ 30%, 250 W high-pressure visible lamp, NCMe	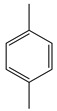	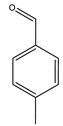	34 ^(a)^	[44]
Nicolae et al.	100 °C, Mn/Fe/O_CIT, TBHP 70%, 24 h, NCMe	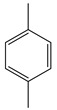	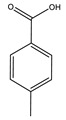	93 ^(b)^	[45]
Ying Lo et al.	30 °C, Cu-MOF, H_2_O_2_ 30%, NCMe, 5 h	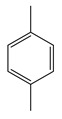	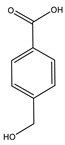	85 ^(b)^	[46]
Xu et al.	150 °C, NCMe, O_2_, Co-BTC + NHPI, 12 h	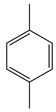	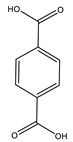	96 ^(b)^	[53]

^(a)^ GC yield; ^(b)^ HPLC yield.

## Abbreviations

2:4-DMP—2,4-dimethylphenol; 2,5-DMP—2,5-dimethylphenol; 4-CBA—4-carboxybelzaldehyde; 4-CTA—crude terephthalic acid; BMIMBr—1-butyl 3-methylimidazolium bromide; BMPA—bis-(2-pyridymethyl)amine; BTC—benzenetricarboxylate; CIT—citrate treatment; DFT—density functional theory; DHMB—di-hydroxymethyl benzene; DMF—dimethylformamide; DMT—dimethyl terephthalate; EPR—electron paramagnetic resonance spectroscopy; HMBA—4-hydroxymethylbenzoic acid; HT—hydrothermal treatment; MCM—Mobil composition of matter; MOF—metal–organic framework; NHPI—*N*-hydroxyphthalimide; PET—polyester terephthalate; PID—proportional-integral-derivative; PX—*p-*xylene; r.t—room temperature; SC—supercritical; TA—*p-*toluic acid; TBHP—tert-butyl hydrogen peroxide; TPA—terephthalic acid; TOF—turnover frequency; XPS—X-ray photoelectron spectroscopy.
